# A comparison of two multiplex-PCR assays for the diagnosis of traveller’s diarrhoea

**DOI:** 10.1186/s12879-021-05885-3

**Published:** 2021-02-16

**Authors:** Frieder Schaumburg, Neele Froböse, Robin Köck

**Affiliations:** 1 Institute of Medical Microbiology, University Hospital Münster, Münster, Germany; 2 DRK Kliniken Berlin, Institute of Hygiene, Berlin, Germany

**Keywords:** Diarrhoea, Travel medicine, Multiplex polymerase chain reaction

## Abstract

**Background:**

Numerous multiplex-PCR assays are now available in routine diagnostics but their clinical value is controversial if a clear association between clinical symptoms and the detection of a particular pathogen is missing. The objective of this work was to evaluate a multiplex-PCR assay for the diagnosis of traveller’s diarrhoea (TD) in a case-control study and to assess the concordance with the BioFire® FilmArray® Gastrointestinal Panel.

**Methods:**

Stool samples from cases (*n* = 61) and controls (*n* = 30) were collected during travel and analysed by the GI-EB Screening assay (Seegene) in a case-control study. The concordance with the BioFire® FilmArray® Gastrointestinal Panel was expressed as the proportion of participants in which both tests agreed in the category “detected” and “not detected”.

**Results:**

None of the test-target organisms (*Campylobacter* spp*., Clostridioides difficile* toxin A/B*, Salmonella* spp.*, Shigella* spp*./*enteroinvasive *Escherichia coli, E. coli* O157*,* Shiga toxin-producing *E. coli, Yersinia enterocolitica)* was significantly associated with TD GI-EB Screening assay. The GI-EB Screening assay had an agreement with the BioFire® FilmArray® of 86.8–100%.

**Conclusion:**

The selection of test-target organisms included in the GI-EB Screening assay appears inappropriate for the diagnostic work-up of TD as none of the detected pathogens was associated with TD. The GI-EB Screening assay had a good concordance with BioFire® FilmArray®.

**Supplementary Information:**

The online version contains supplementary material available at 10.1186/s12879-021-05885-3.

## Background

The World Health Organization (WHO) defines diarrhoea as “the passage of three or more loose or liquid stools per day, or more frequently than is normal for the individual” [1]. Among travel related morbidity, traveller’s diarrhoea (TD) is in first place [2]. The causative agents of TD are manifold (e.g. virus, bacteria, parasites) and often remain unidentified in culture-based routine microbiological analysis. Recently, various culture-independent syndromic multiplex assays were developed for the laboratory detection of a broad range of TD pathogens. Although these tests are more sensitive, their interpretation can become a challenge, if numerous pathogens are detected [3]. In addition, some pathogens have a weak association with disease or low attributable fractions among TD cases (e.g. *Aeromonas* sp., *Plesiomonas shigelloides*) [4, 5]. Finally, the presence of amplifiable DNA does not always correlate with the presence of viable pathogens.

As molecular tests for gastrointestinal infections become more and more part of routine diagnostics [6], there is a need to assess their clinical value for the diagnosis of TD. Therefore, the objective of this study was to evaluate the GI-EB Screening assay (Allplex™, Seegene, Düsseldorf, Germany) in a case-control study.

## Methods

### Stool samples

This study made use of an already existing collection of samples from international travellers with (*n* = 61) and without TD (*n* = 30) that were collected between 2016 and 2018 [5]. Age ≥ 18 years was the only inclusion criterion; no exclusion criteria were applied. Cases were defined according to the WHO-definition of diarrhoea [1]. Samples were collected and stored during travel in Cary-Blair medium (Faecal Transwab® Check Diagnostics, Westerau, Germany) and the first diarrheagenic sample from cases was included in the study [7]. Controls were randomly selected from asymptomatic travellers.

The samples were originally analysed for the acquisition dynamics of antimicrobial resistant bacteria (e.g. ESBL-producing Enterobacterales, vancomycin-resistant enterococci, carbapenem- or colistin-resistant Gram-negative bacteria) during international travel [7]. This set was also screened by culture for *Clostridioides difficile* (not detected), but not for any other enteropathogen.

### Multiplex-PCR assay

DNA was extracted using GenoXtract (Hain, Nehren, Germany). The GI-EB Screening multiplex real-time PCR was performed on a CFX96 thermal cycler (Biorad, Feldkirchen, Germany) to detect *Campylobacter* spp*., Clostridioides difficile* toxin A/B*, Salmonella* spp.*, Shigella* spp*./*enteroinvasive *Escherichia coli* (EIEC)*, E. coli* O157*,* Shiga toxin-producing *E. coli* (STEC) and *Yersinia enterocolitica* according to the manufacturer’s instruction. Amplification curves were evaluated with Seegene viewer (V3.18.003). The same set of stool samples was already tested using the BioFire® FilmArray® Gastrointestinal Panel (bioMérieux, Marcy l’Étoile, France) [5].

### Statistics

We compared categorical variables (e.g. the proportions of positive test results) between both groups (with and without TD) using Chi^2^-Test or Fisher’s exact test when appropriate and calculated the Odds-ratios (OR) and 95% confidence interval (95% CI). The OR was used to compute the attributable fraction (AF = proportion of a pathogen in the case group • [1 − (1/OR)]) [4]. The concordance between results from the GI-EB Screening assay and BioFire® FilmArray® Gastrointestinal Panel was calculated as the proportion of participants in which both tests agreed in the category “detected” and “not detected”.

### Cost calculation

The overall costs of the GI-EB Screening assay and BioFire® FilmArray® Gastrointestinal Panel were calculated considering both costs for consumables and work force. Cost for work force were based on the sum of hands-on-time and the salary scale of laboratory technicians in the public service in Germany (4.250 €/month). In contrast to the GI-EB Screening assay, the BioFire® FilmArray® Gastrointestinal Panel does not require additional consumables for controls. Costs for controls were not included in this calculation as it depends on how many samples were processed in parallel.

## Results

A detailed description of the study population (*n* = 91), the cases (*n* = 61) and controls (*n* = 30) is published elsewhere [5]. Briefly, the TD cases were younger (mean age 24 vs. 39, *p* < 0.001) and more likely female (66 vs. 33%, *p* = 0.004) than controls. The majority of participants travelled to Africa (36%), Asia (34%) and North America (15%). The travel destinations were unbalanced between cases and controls as more cases travelled to Africa (OR = 2.5, 95%CI: 0.9–5, *p* = 0.07) while a travel destination in South America was overrepresented in the control group (OR = 5, 95%CI: 1.4–25, *p* = 0.01).

The average duration of signs and symptoms of TD was 4 days. Stool samples were stored at ambient temperature during travel and at − 20 °C after travel until analysis (storage time at − 20° was approx. 2 years).

In total, 29% of participants (26/91) were tested positive in the GI-EB Screening assay. The majority had only one pathogen (20/91), followed by two pathogens (4/91) and three or four pathogens (each 1/91).

Overall, *E. coli* O157 was predominant (13/91), followed by STEC (9/91), *Shigella* spp./EIEC (5/91), *Y. enterocolitica* (5/91) and *Salmonella* spp. (3/91). Noteworthy, three travellers (2 cases, 1 control) had a co-detection of *E. coli* O157/STEC. Although *Salmonella* spp. was only detected in cases, none of the pathogens included in the GI-EB Screening assay was significantly associated with TD cases. All AFs were 0–3.5 (Table 1).

**Table 1:** Evaluation of the GI-EB Screening assay (Seegene) in travellers with and without traveller’s diarrhoea (TD)

Pathogen	Total (*n* = 91) [n (%)]	Cases (travellers with TD, *n* = 61) [n (%)]	Controls (travellers without TD, *n* = 30) [n (%)]	OR (95%CI)	*p*-value	AF (95%CI)
*Escherichia coli* O157	13 (14.3%)	8 (13%)	5 (17%)	0.8 (0.2–2.5)	0.65	0 (0–7.8)
Shiga-toxin producing *E. coli*	9 (9.9%)	4 (7%)	5 (17%)	0.4 (0.1–1.4)	0.15	0 (0–2.0)
*Shigella* spp./Enteroinvasive *E. coli*	5 (5.5%)	4 (7%)	1 (3%)	2 (0.2–10)	1	3.5 (0–6.3)
*Yersinia enterocolitica*	5 (5.5%)	3 (5%)	2 (7%)	0.7 (0.1–5)	1	0 (0–4)
*Salmonella* spp.	3 (3.3%)	3 (5%)	0 (0%)	0 (0–NaN)	0.55	NA
*Clostridioides difficile* toxin A/B	0 (0%)	0 (0%)	0 (0%)	NA	NA	NA
*Campylobacter* spp.	0 (0%)	0 (0%)	0 (0%)	NA	NA	NA

Note: *NA* (not applicable), *NaN* (not a number), *AF* (attributable fraction), *OR* (Odds ratio), *TD* (Traveller’s diarrhoea), *95%CI* (95% confidence interval)

A higher pathogen load corresponds to a lower Ct-value (threshold cycle, i.e. number of cycles required for a positive result) and could be used to distinguish between asymptomatic colonization and TD (e.g. ETEC, *Campylobacter*) assuming that pathogen concentration is higher in cases compared to controls [4]. The mean Ct-values were significantly higher in cases compared to controls for *E. coli* O157 (37.7 vs. 32.9, *p* = 0.03). Cases and controls had comparable Ct-values for *Y. enterocolitica* (34.3 vs. 33.6, *p* = 0.9) and STEC (35.6 vs. 34.9, *p* = 0.8). No comparison of Ct-values was done for *Salmonella* spp. and *Shigella* spp./EIEC as none or only one participant, respectively, was detected in the control group (Table 1).

The samples were also analysed by the BioFire® FilmArray® Gastrointestinal Panel for comparison [5] and the majority of pathogens were enteropathogenic *E. coli* (36/91, Table S1).

The concordance between the GI-EB Screening assay and BioFire® FilmArray® Gastrointestinal Panel was 86.8–100% depending on the target pathogen (Table 2).

**Table 2:** Concordance between GI-EB Screening assay (Seegene) and BioFire® FilmArray® Gastrointestinal Panel (bioMérieux [5])

			GI-EB Screening assay (Seegene)		Concordance [% [n/n)]
			Not detected [n]	Detected [n]	
BioFire® FilmArray® Gastrointestinal Panel	*Escherichia coli* O157	Not detected	76	6	91.2% (83/91)
		Detected	2	7	
	Shiga-toxin producing *E. coli*	Not detected	70	0	86.8% (79/91)
		Detected	12	9	
	*Shigella* spp./Enteroinvasive *E. coli*	Not detected	85	1	97.8% (89/91)
		Detected	1	4	
	*Yersinia enterocolitica*	Not detected	86	3	94.5% (88/91)
		Detected	0	2	
	*Salmonella* spp.	Not detected	88	0	100% (91/91)
		Detected	0	3	

Total costs for one analysis using the BioFire® FilmArray® Gastrointestinal Panel were 118.15 € (work force costs for 10 min: 3.15 €; consumable costs: 115.00€). One test with the GI-EB Screening assay costs 25.43 € (work force costs for 20 min: 6.30 €; consumable costs [incl. DNA extraction]: 19.13 €).

## Discussion

We tested a selection of TD stool samples with the GI-EB Screening assay and found a low proportion of positive samples and a weak association with TD (Table 1). This weak test performance is most likely due to unsuitable target organisms of the test for the diagnosis of TD (Table 1). In general, ETEC (7–45%), enteropathogenic *E. coli* (EPEC, 26–47%) and enteroaggregative *E. coli* (EAEC, 5–46%) are predominant in TD in many countries [2, 8].

Ct-values were significantly higher in cases compared to controls for *E. coli* O157 suggesting a higher pathogen load in controls. The reason for this finding is, however, unclear and in contradiction with a larger study showing that the association with TD increases with lower Ct-values for STEC [4].

The good concordance (86.8–100%) of the GI-EB Screening assay with BioFire® FilmArray® Gastrointestinal Panel is in line with another report that showed a concordance of 82.6–100% for bacterial pathogens between GI-EB Screening assay and other multiplex platforms that are currently used (e.g. BioFire®, Luminex xTAG®) [9].

Syndromic multiplex tests are currently very much in vogue but their clinical value is often blurred due to the lack of asymptomatic control groups in the majority of studies [10, 11]. Without this control group, one cannot calculate the AF, which is a benchmark in the evaluation of these multiplex tests as it “indicates the proportion of cases that can be attributed to a particular pathogen” [2, 4]. Increasing detection rates or sensitivity does not imply an additional clinical value [11]. We therefore suggest that future studies must include an asymptomatic control group to shed light on the question, which detected pathogen is truly of clinical relevance.

The total costs (personnel and reagents) were markedly lower for the GI-EB Screening assay (25.43 €) than the BioFire® FilmArray® Gastrointestinal Panel (118.15 €) and comparable to other commercial PCR-kits covering similar bacterial species (e.g. RIDA®GENE: 20–25 € without DNA extraction) [12]. The more affordable PCR-kits, however, require a more sophisticated infrastructure (e.g. DNA extraction, test-platforms) and are suitable for processes in laboratories. In contrast, the more expensive BioFire® FilmArray® Gastrointestinal Panel has its strength as a point-of-care diagnostics.

Our study has limitations: First, the small sample size that does not allow for any conclusions on an association between the detection of rare pathogens and TD (e.g. *Salmonella*, *Campylobacter*). Second, we were unable to compare the multiplex-approaches with classical culture-based microbiological analyses, which was not done right after the return of the travellers. A *post-hoc* culture for enteropathogens more than two years after collection would lead to an unacceptably high rate of false-negative results by culture, particularly for fastidious pathogens such as *Campylobacter* sp. Third, we initially tested if sufficient controls are in our dataset to match them with the travel region of TD cases to rule out geographic confounders. Since we were unable to include matched controls for each case, we decided to select controls randomly. Therefore, the absence of an association between the detection of pathogens and TD might be confounded.

## Conclusion

The GI-EB Screening assay is not suitable for the analyses of TD, as relevant target bacteria are not included in the assay and those included in the assay show very poor association with TD cases. The concordance between the GI-EB Screening assay and BioFire® FilmArray® Gastrointestinal Panel is good.

## Supplementary Information

**Additional file 1: Table S1**: Test results of the BioFire® FilmArray® Gastrointestinal Panel as reported elsewhere [1].

## Data Availability

The datasets used and/or analysed during the current study are available from the corresponding author on reasonable request.

## Abbreviations

AF: Attributable fraction
EAEC: Enteroaggregative *Escherichia coli*
EPEC: Enteropathogenic *Escherichia coli*
ESBL: Extended-spectrum betalactamases
EIEC: enteroinvasive *Escherichia coli*
OR: Odds-ratios
STEC: Shiga toxin-producing *Escherichia coli*
TD: Ttraveller’s diarrhoea
WHO: World Health Organization
95% CI: 95% confidence interval
