# Supplementary material for: A comparison of two multiplex-PCR assays for the diagnosis of traveller’s diarrhoea
Source: BMC Infect Dis. 2021 Feb 16;21:181. doi: 10.1186/s12879-021-05885-3 (PMC7888160; doi:10.1186/s12879-021-05885-3)
Supplement: Supplementary file 1 — Additional file 1: Table S1: Test results of the BioFire® FilmArray® Gastrointestinal Panel as reported elsewhere [1]. [file 12879_2021_5885_MOESM1_ESM.docx]

**Research article**

**Title:**

A comparison of two multiplex-PCR assays for the diagnosis of traveller’s diarrhoea

**Authors:**

Frieder Schaumburg^a,#^, Neele Froböse^a^, Robin Köck^b^

**Affiliation:**

^a^Institute of Medical Microbiology, University Hospital Münster, Münster, Germany

^b^DRK Kliniken Berlin, Institute of Hygiene, Berlin, Germany

^#^Corresponding author: [frieder.schaumburg@ukmuenster.de](mailto:frieder.schaumburg@ukmuenster.de)

Table S1: Test results of the BioFire^®^ FilmArray^®^ Gastrointestinal Panel as reported elsewhere (1)

|  | Total (n=91) [n (%)] | Cases (travellers with TD, n=61) [n (%)] | Controls (travellers without TD, n=30) [n (%)] |
| --- | --- | --- | --- |
| *Escherichia coli* O157 | 9 (10%) | 3 (5%) | 6 (20%) |
| Shiga-toxin producing *E. coli* | 21 (23%) | 10 (16%) | 11 (37%) |
| *Shigella* spp./Enteroinvasive *E. coli* | 5 (6%) | 4 (7%) | 1 (3%) |
| *Yersinia enterocolitica* | 2 (2%) | 0 (0%) | 2 (7%) |
| *Salmonella* spp. | 3 (3%) | 3 (5%) | 0 (0%) |

References

1. Schaumburg F, Correa-Martinez CL, Niemann S, Köck R, Becker K. Aetiology of traveller's diarrhea: A nested case-control study. Travel medicine and infectious disease. 2020:101696.
